# Investigating the host specificity of *Campylobacter jejuni* and *Campylobacter coli* by sequencing gyrase subunit A

**DOI:** 10.1186/s12866-014-0205-7

**Published:** 2014-08-28

**Authors:** Catherine Ragimbeau, Stephanie Colin, Anthony Devaux, Frédéric Decruyenaere, Henry-Michel Cauchie, Serge Losch, Christian Penny, Joël Mossong

**Affiliations:** National Health Laboratory, Surveillance and Epidemiology of Infectious Diseases, 1 rue Louis Rech, Dudelange, L-3555 Luxembourg; Centre de Recherche Public Santé, 1A-B rue Thomas Edison, Strassen, L-1445 Luxembourg; National Health Laboratory, Bacteriology- Parasitology- Mycology, 1 rue Louis Rech, Dudelange, L-3555 Luxembourg; Département Environnement et Agro-Biotechnologies, Centre de Recherche Public – Gabriel Lippmann, 41 rue du Brill, Belvaux, L-4422 Luxembourg; Veterinary Medecine Laboratory, 54, av. Gast Diderich, Luxembourg, L-1420 Luxembourg

**Keywords:** *Campylobacter jejuni*, *Campylobacter coli*, Gyrase, Typing, MLST, GyrA, Surveillance, Surface water

## Abstract

**Background:**

Surveillance and field investigations of *Campylobacter* infections require molecular tools with genetic markers appropriate for tracing purposes, *i.e.* based on the principle that some *Campylobacter* lineages acquire a host signature under adaptive selection pressure. We developed a sequence-based method targeting the quinolone resistance determining region within the subunit A of DNA gyrase (*gyrA*). Host specificity was evaluated by characterizing two collections of *Campylobacter jejuni* (N = 430) and *Campylobacter coli* (N = 302) originating from surface waters, domestic mammals and poultry.

**Results:**

Based on nucleotide identity, a total of 80 *gyrA* alleles were observed. Thirty nine alleles assigned to *C. coli* encoding two peptides fell into three clades: two associated with surface waters and one associated with domestic mammals and poultry. The variability in GC content generated by synonymous mutations suggested that surface waters isolates originated from two distinct ecological niches. A total of 42 alleles were recorded from C*. jejuni* strains and encoded 8 peptides including one lying in a distinct lineage associated with wildlife. Seven of the 23 alleles encoding peptide #1 displayed the synonymous mutation G408A not identified in poultry isolates. By contrast, the substitution Ser22Gly observed in 4 different peptide groups was significantly associated with domestic birds (*P* = 0.001). The change in amino acid sequences Thr86Ile conferring resistance to quinolones was significantly associated with poultry (*P* < 0.001) in both *C. jejuni* and *C. coli* with 38.7% and 67.9% of quinolone-resistant strains, respectively.

**Conclusions:**

The *gyrA* typing method presented here is an informative tool as sequences appear to be predictive of particular ecological niches. Combined with multi-locus sequence typing, it could increase the resolution of source attribution, and combined with *porA*/*flaA* typing it could be suitable for detecting temporal clusters of human cases. All *gyrA* alleles identified were deposited in the freely accessible online database http://pubmlst.org/campylobacter.

**Electronic supplementary material:**

The online version of this article (doi:10.1186/s12866-014-0205-7) contains supplementary material, which is available to authorized users.

## Background

According to the EU Summary Report 2013, *Campylobacter* infections have superseded *Salmonella* infections in many Member States as the most frequently reported food-borne infection, and many countries have been witnessing recent increases in reported cases [1]. In 2011, the incidence rate in Luxembourg has increased to 138 per 100,000 population, which is a national record and among the highest in Europe [1]. As a result, the competent national authorities in Luxembourg have recognized the rising trend of *Campylobacter* infections as a national public health priority [2]. Approximately 80 to 90% of the human cases is caused by the species *C. jejuni* and the remainder is primarily caused by *C. coli*. While exposure to contaminated food (and in particular chicken) is thought to be the most important route of transmission of campylobacteriosis, several studies in Europe have indicated that environmental routes of transmission could be important [3-5].

As a complimentary approach to classical epidemiology (e.g. measuring food intake and other exposures), molecular epidemiology has proved very useful for investigating likely sources of *Campylobacter* infections [6-9]. However, predicting the biological host from the genotype is challenging because *Campylobacter* species display a weak clonal population structure, in which the different lineages and the relatedness between isolates cannot be easily determined. The multilocus sequence typing (MLST) method exploits the relative conservation in sequence of 7 core genes encoding housekeeping functions in which variations are more likely to be selectively neutral [10]. This approach is now recognized as the gold standard typing method for this bacteria genus but for short-term epidemiology like cluster detection or for tracing transmission routes in a defined space-time window, MLST should be combined with other markers to increase the discrimination power of the typing scheme. For that purpose, the loci encoding the flagellin *flaA*, *flaB* and the variable outer membrane protein *porA* were proposed [8].

In addition to these genotypic aspects, a phenotypic trait related to fluoroquinolone resistance has become of major epidemiologic relevance. Indeed, about half of *C. jejuni* isolated from humans in Europe are resistant to ciprofloxacin, an antimicrobial often used for treating severe foodborne infections. Since *Campylobacter* is a zoonotic bacterium, the emergence of resistant strains has been linked to a selective pressure generated by the extensive use of quinolones in food-producing animals [11]. Enrofloxacin is one of the major fluoroquinolone agents for prophylactic or therapeutic veterinary purposes. In poultry production, the whole flock is generally treated by adding this compound to the drinking water, whereas, in cattle or pig production, treatment is often restricted to diseased animals. As a result, the highest levels of quinolone resistance are found in *Campylobacter* isolated from chicken (*Gallus gallus*) [12]*.* Fluoroquinolones are categorized as critically important drugs for human medicine by the WHO [13], and consequently surveillance programs to monitor trends in use [14] and resistance [15,16,12] have been implemented. For *Campylobacter*, the principal molecular mechanisms of quinolone resistance consists in a single mutation C257T in the *gyrA* gene [17,18]. Consequently, PCR or sequenced-based methods targeting this quinolone resistance determining region (QRDR) have been shown to be highly predictive for detecting phenotypically resistant variants [16]. Moreover, previous work on *gyrA* suggested this locus might provide a host signature and thus be a good candidate for typing purposes [19,20].

The aims of this study were thus to evaluate the host specificity of the *gyrA* gene and to monitor quinolone resistance in a large *Campylobacter jejuni* and *coli* strain collection originating from domesticated animals and surface water samples potentially contaminated by wildlife.

## Methods

### Isolates from non-human sources

For this study, we characterized 430 *C. jejuni* and 280 *C. coli* isolated in Luxembourg from surface waters (SW), domesticated mammals (DM) and poultry (P) between 2005 and 2012. Identification to the species level of the isolates was previously achieved by a duplex real-time PCR targeting the *hip*O gene of *C. jejuni* and a conserved region of the *gyr*A gene of *C. jejuni* and *C. coli* (outside the QRDR). Primer and probe combinations for the *hip*O Taqman-qPCR and *gyr*A FRET-qPCR systems were selected from published methods [21,22]. Real-time PCRs were performed using the FastStart DNA Master^plus^ HybProbe kit (Roche Diagnostic, Prophac, Luxembourg) in a total reaction volume of 20 μl containing the following final primer and probe concentrations: *hip*O primers 0.5 μM, *hip*O Taqman probe 0.1 μM, *gyr*A primers 1 μM and *gyr*A sensor and anchor probes 0.2 μM. The PCR programme included an initial activation step of 10 min at 95°C, 30 amplification cycles of 6 s at 95°C, 12 s at 54°C and 25 s at 72°C, followed by a melting curve analysis step of 1 min at 95°C, 50 s at 38°C, a rise to 80°C with an increase rate of 0.1°C s^−1^, and final cooling of 30 s at 40°C. *C. jejuni* and *C. coli* were identified by reading both the amplification and melting curves. Isolates with an atypical profile (i.e. *hipO* negative and a *gyrA* melting curve corresponding to no known species) were further confirmed as *C. jejuni* with a conventional agarose gel-based PCR targeting the *hipO* gene with a new set of primers designed for this study: hipO-58 F 5’CAAATTCATGAAAATCCTG 3’ and hipO1057R 5’TGTCGTTTTCATTTTCTAA 3’.

DM isolates were obtained from faeces while P isolates were obtained from raw meat and faeces. Because only few local *C. coli* isolates of pig origin were available for analysis (N = 23), we characterized as part of the DM collection further 22 porcine *C. coli* strains from collections from France (N = 16, year 2008) and Belgium (N = 6, year 2010).

A total of 31 SW sites were sampled from different geographic areas in Luxembourg (surface 2,586 km^2^) including rivers, pond waters, recreational waters and wastewater treatment plant outlets between January 2011 and December 2012. The SW *C. jejuni* (N = 206) and *C. coli* (N = 123) isolates were obtained from 23 and 22 different water sites, respectively, and both species were simultaneously obtained from 14 sites.

The *C. jejuni* collection included 99 DM isolates (bovine, N = 81; dog, N = 6; ovine, N = 4; equidae, N = 4; goat, N = 3; cat, N = 1) and 125 P isolates (broiler, N = 94; turkey, N = 19, duck, N = 8; quail, N = 3, ostrich, N = 1). The *C. coli* collection included 46 DM isolates (pig, N = 45; goat, N = 1) and 133 P isolates (broiler, N = 104; turkey, N = 25; duck, N = 1; guinea fowl, N = 1, quail, N = 1; ostrich, N = 1). All isolates were stored in FBP medium [23] at −70°C until use.

### DNA isolation

Isolates were subcultured on chocolate PolyVitex agar (ref 42079, Biomérieux, France) at +42°C for 24 h in a microaerobic atmosphere (6% O_2_, 3.6% CO_2_, 3.6% H_2_ and 86.9% N_2_) generated by an Anoxomat™ system (Mart Microbiology, Belgium). Bacterial DNA was extracted from these cultures with the DNA QIAamp mini Kit 250 (ref 51306, Qiagen, The Netherlands). From stock solutions, tenfold dilutions in buffer AE (10 mM Tris · Cl; 0.5 mM EDTA; pH 9.0) were prepared for the PCR assays.

### *gyrA* sequencing

The partial gene sequence of *gyrA* targeting the quinolone resistance determining region (QRDR) was amplified and sequenced with the forward primer GYR-for (5’-GCTGATGCAAAAGKTTAATATGC-3’) and the reverse primer GYR-rev (5’-TTTGTCGCCATACCTACAGC-3’) designed for this study. Amplifications were carried out in a total volume of 20 μl using the AmpliTaq Gold 360 Master Mix (code 4398901, Applied Biosystems, Belgium). The primer concentration was adjusted at 0.2 μmol l^−1^ each in the reaction mix and the cycling conditions were as follows: 95°C for 10 min then 35 cycles of 95°C 30 s, 55°C 30 s, 72°C 50 s. The reaction was completed by a final extension of 5 min at 72°C. For the sequencing step, the PCR products were diluted ten-fold in water and the sequencing reaction was carried out directly with 2 μl from these dilutions. The sequencing reactions were purified by the Agencourt® CleanSEQ® method (Protocol 000411v001, Beckman Coulter, USA) and products were analyzed with an ABI Prism 3130XL sequencer (ABI, Life Technologies, Belgium).

An in-house nomenclature was determined for the assignment of the nucleotide and peptide sequences (length analyzed = 496 bp corresponding to 165 aa): numbering of the alleles of *C. coli* started at #301. All the sequences identified and assigned were included in the online database *Campylobacter* Multi Locus Sequence Typing [24] and sequence query was done by selecting the loci named fn_gyrA and fp_gyrA (for nucleotide alleles and peptide sequences, respectively). The number assignment of alleles was based on a larger strain collection than the one presented herein, such that not all allele numbers are represented in this study.

### Multi Locus Sequence Typing (MLST)

The MLST protocol for amplification and sequencing of the seven housekeeping genes developed by Dingle *et al.* was used for this study [25,26]. Sequencing steps were carried out as described earlier (dilution of the PCR amplicons in water, use of magnetic beads for purification of the sequence reactions). Automated data analysis and library matching were set up with SeqScape® software v2.5 (ABI, Life Technologies, Belgium). New alleles and STs identified were submitted for assignment to the MLST database [24].

### Data analysis

The START 2 program [27] was used for: (i) calculating the index of association (I_A_), reflecting the degree of clonality in each population (SW, DM and P), from allelic profiles generated by MLST and *gyrA* data combined; (ii) determining the ratio of non-synonymous (*d*_N_) to synonymous (*d*_S_) substitutions per nucleotide site in the *gyrA* sequence. The index of population differentiation (*F* statistic, denoted *F*_ST_) was estimated using Arlequin, v3.1 program [28] from the concatenated sequences of the 8 loci (MLST combined with *gyrA*). An *F*_ST_ of 0 indicates that two populations are indistinguishable, whereas an *F*_ST_ value of 1 indicates that two populations are genetically distinct. The discriminating power of the molecular methods (MLST, *gyrA* sequencing) were estimated by the Simpson's Index of Diversity (SID) applied to the test population and calculated with the freely available online tool Comparing Partitions [29,30]. The SID measures the probability that two individuals selected at random belong to the same genotype. Alignment of *gyrA* sequences and calculation of GC content (%) was performed with BioEdit v7.0.5.3 [31]. The neighbour-joining radial tree was constructed using MEGA 5 [32] with the *gyrA* sequences from all the alleles identified in both species. The robustness of the nodes was evaluated by bootstrapping (200 replicates). Normal distribution verification and unpaired two-sample t-test comparisons on mean GC percentages between *gyrA* clusters were done using the GraphPad Prism software tool.

## Results

### *gyrA* sequencing data

**Figure 1:**
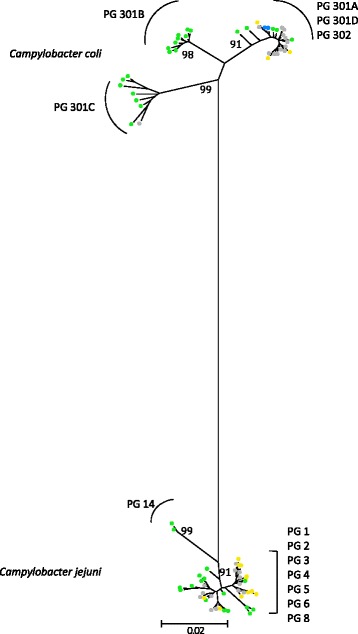
**Neighbour-joining radial distance phylogenetic tree constructed with the 80 nucleotide** ***gyrA*** **alleles identified.** PG = peptide group. Bootstrap support values (%) for each of the nodes leading to the *gyrA* sequence clusters are indicated. Key: surface waters, green; domesticated mammals, blue; poultry, yellow; multi-source, grey.

With the primers designed in this study, amplification and partial sequencing of *gyrA* was successfully performed for all strains tested in both species *C. jejuni* and *C. coli.* An overall total of 80 different nucleotide alleles were identified. Alignment of the sequences revealed two main allelic groups, sharing overall 81.3% nucleotide sequence identity. A first group of 41 alleles contained all but one *C. jejuni* isolates (99.8% of the *C. jejuni* collection). A second group of 39 alleles contained all but 7 *C. coli* isolates (97.7% of the *C. coli* collection). Interestingly, the 39 alleles related to *C. coli* encode only two different peptide sequences that differ in one single amino acid (Thr86Ile substitution giving rise to quinolone resistance). By contrast, the 41 alleles related to *C. jejuni* encode 8 different peptide sequences (numbered between #1 and #14). The *d*_N_/*d*_S_ ratios were lower for the *C. coli* (0.0075) than the *C. jejuni* (0.0498) collections, reflecting a higher level of synonymous changes within the *gyrA* sequences of the *C. coli* than in those of *C. jejuni.* The phylogenic tree in Figure 1 further highlights two clades for *C. jejuni* and three clades for *C. coli*.

### Genetic diversity among the *gyrA* sequences within each species

The nucleotide sequences were aligned to an arbitrarily chosen reference allele (allele #1 and #301 for *C. jejuni* and *C. coli*, respectively). A total of 36 and 46 polymorphic sites were found for *C. jejuni* and *C. coli,* respectively. Next, nucleotide alleles were classified in a two-step approach: first, according to the encoded peptide (i.e. non-synonymous mutations) and second, according to similarities in nucleotide sequences (i.e. synonymous mutations). Tables 1 and 2 display this classification and show a selection of synonymous and non-synonymous changes in sequences that were common to several alleles and which determined different peptide groups (PG). The 430 isolates of the *C. jejuni* collection were classified into 9 PGs: 8 corresponded to PGs #1, 2, 3, 4, 5, 6, 8 and 14 related to *C. jejuni* (41 nucleotide alleles) and one corresponded to PG #301 related to *C. coli* (encoded by the nucleotide allele #301, Table 1). For refining grouping among the 302 *C. coli* strains, PG #301 (originally composed of 39 nucleotide alleles) was subdivided in four parts named A, B, C and D according to similarities in synonymous mutations in their nucleotide sequences (Table 2). PG #302 included all strains with the quinolone resistance C257T mutation (10 nucleotide alleles). The remaining peptide groups were specific to the *C. jejuni* species (PGs #7, 8, 9 and 23).

**Table 1:** **Distribution of** ***C. jejuni gyrA*** **alleles by source and conserved nucleotide**

**Peptide group**	Allele no.*	**Nucleotide position**									Distribution by source**			**No. of ST**
		**64**	**111**	**210**	**257**	**276**	**324**	**408**	**438**	**486**	**SW**	**DM**	**P**	
	1	A	G	C	C	G	A	G	C	A	26	27	22	26
	4	.	.	.	.	.	.	.	.	.	2	14	6	6
	5	.	.	.	.	.	.	.	.	.	3	12	10	11
	7	.	.	.	.	.	.	.	.	.	45	8	16	11
	11	.	.	.	.	.	.	A	.	.	26	10		22
	12	.	.	.	.	.	.	.	.	.			1	1
	13	.	.	.	.	.	.	.	.	.	3		4	5
	16	.	.	.	.	.	.	.	.	.			4	2
	18	.	.	.	.	.	.	.	.	.			1	1
	19	.	.	.	.	.	.	.	.	.	6			1
	31	.	.	.	.	.	.	.	.	.	22	1		11
1	36	.	.	.	.	.	.	.	.	.	5			5
	39	.	.	.	.	.	.	A	.	.	1			1
	40	.	.	.	.	.	.	A	.	.	13			8
	41	.	.	.	.	.	.	A	.	.	3			3
	56	.	.	.	.	.	.	A	.	.	3			2
	66	.	.	.	.	.	.	A	.	.	1			1
	73	.	.	.	.	.	.	.	.	.	1			1
	74	.	.	.	.	.	.	.	.	.	1			1
	75	.	.	T	.	.	.	.	.	.	1			1
	76	.	.	.	.	.	.	.	.	.	2			1
	79	.	.	.	.	.	.	A	.	.	1			1
	80	.	.	.	.	.	.	.	.	.	1			1
	2	.	.	.	T	.	.	.	.	.			3	3
	3	.	.	.	T	.	.	.	.	.	9	3	6	9
	8	.	.	.	T	.	.	.	.	.	14	17	13	14
2	15	.	.	.	T	.	.	.	.	.			2	2
	17	.	.	.	T	.	.	.	.	.			2	2
	30	.	.	.	T	.	.	.	.	.	3		1	4
	44	.	.	.	T	.	.	.	.	.			2	2
3	6	G	.	.	.	.	.	.	.	.			1	1
	9	G	.	.	T	.	.	.	.	.	2	2	20	11
4	53	G	.	.	T	.	.	.	.	.	1			1
	78	G	.	.	T	.	.	.	.	.			1	1
	10	G	.	.	.	.	.	.	.	.	7	4	6	10
5	23	G	.	.	.	.	.	.	.	.			1	1
	27	G	.	.	.	.	.	.	.	.	1			1
6	14	.	.	.	.	.	.	.	.	.			1	1
8	24	G	.	.	T	.	.	.	.	.		1	1	2
14	54	.	A	T	.	A	G	A	T	G	1			1
	55	.	A	T	.	A	G	A	T	G	2			1
301	301	.	T	T	.	A	.	.	A	.			1	1

*Nucleotide allele number, **SW = Surface water, *DM* = Domesticated Mammals, P = Poultry.

**Table 2:** **Distribution of** ***C. coli gyrA*** **alleles by source and conserved nucleotide**

Peptide group*	**Allele no.**	**Nucleotide position**																				Distribution by source**			**No. of ST**
		**21**	**69**	**78**	**81**	**90**	**144**	**177**	**180**	**195**	**257**	**267**	**273**	**276**	**279**	**300**	**414**	**417**	**435**	**477**	**495**	**SW**	**DM**	**P**	
	301	A	T	T	T	C	C	C	A	A	C	C	C	A	A	T	A	C	G	C	G	1	2	9	11
	308	.	.	.	.	.	.	.	.	.	.	.	.	.	.	.	.	.	.	.	.	4	2	14	10
	309	.	.	.	.	.	.	.	.	.	.	.	.	.	.	.	.	.	.	.	.	2	11	1	8
301 A	312	.	.	.	.	.	.	.	.	.	.	.	.	.	.	.	.	.	.	.	.	1	1	2	4
	316	.	.	.	.	.	.	.	.	.	.	.	.	.	.	.	.	.	.	.	.	4	10	10	18
	321	.	.	.	.	.	.	.	.	.	.	.	.	.	.	.	.	.	.	.	.		2		1
	318	.	.	.	.	.	.	.	.	.	.	.	.	.	.	.	.	.	.	.	.	5	5	5	8
	323	.	.	.	.	.	.	G	.	.	.	T	.	G	.	A	G	T	.	T	.	16			10
	324	.	.	.	.	.	.	G	G	.	.	T	.	G	.	A	G	T	.	T	.	1			1
	325	.	.	.	.	.	.	G	.	.	.	T	.	G	.	A	G	T	.	T	.	13			10
	327	.	.	.	.	.	.	G	.	.	.	T	.	G	.	A	G	T	.	T	.	6			3
301 B	334	.	.	.	.	.	.	G	.	.	.	T	.	G	.	A	G	T	.	T	.	2			2
	342	.	.	.	.	.	.	G	.	.	.	T	.	G	.	A	G	T	.	T	.	2			1
	346	.	.	.	.	.	.	G	.	.	.	T	.	G	.	A	G	T	.	T	.	2			2
	349	.	.	.	.	.	.	G	.	.	.	T	.	G	.	A	G	T	.	T	.	1			1
	350	.	.	.	.	.	.	G	.	.	.	T	.	G	.	A	G	T	.	.	.	1			1
	314	G	C	C	C	T	T	.	G	G	.	T	T	G	G	A	G	T	A	T	A	1		1	1
	329	G	C	C	C	T	T	.	G	G	.	T	T	G	G	A	G	T	A	T	A	1			1
	330	G	C	C	C	T	T	.	G	G	.	T	T	G	G	A	G	T	A	T	A	2			2
301 C	331	G	C	C	C	T	T	.	G	G	.	T	T	G	G	A	G	T	A	T	A	1			1
	336	G	C	C	C	T	T	.	G	G	.	T	T	G	G	A	G	T	A	T	A	3			3
	343	G	C	C	C	T	T	.	G	G	.	T	T	G	G	A	G	T	A	T	A	1			1
	345	G	C	C	C	T	T	.	G	G	.	T	T	G	G	A	G	T	A	T	A	1			1
	348	G	C	C	C	T	T	.	G	G	.	T	T	G	G	A	G	T	A	T	A	1			1
	320	.	.	.	.	.	T	.	.	.	.	.	.	.	.	.	.	.	.	.	.	1			1
	322	.	.	.	.	.	.	.	.	.	.	.	.	.	.	.	.	.	.	T	.	9			4
301 D	332	.	.	.	.	.	T	G	.	.	.	T	.	.	.	.	.	.	A	.	.	7			1
	335	.	.	.	.	.	.	.	.	.	.	.	.	.	.	.	.	.	A	.	.	4			3
	337	.	.	.	.	T	.	.	.	.	.	.	.	.	.	.	.	.	.	.	.	1			1
	302	.	.	.	.	.	.	.	.	.	T	.	.	.	.	.	.	.	.	.	.	6	5	6	11
	303	.	.	.	.	.	.	.	.	.	T	.	.	.	.	.	.	.	.	.	.			1	1
	304	.	.	.	.	.	.	.	.	.	T	.	.	.	.	.	.	.	.	.	.	2		9	6
	305	.	.	.	.	.	.	.	.	.	T	.	.	.	.	.	.	.	.	.	.	8		21	15
	306	.	.	.	.	.	.	.	.	.	T	.	.	.	.	.	.	.	.	.	.	6	1	33	23
302	310	.	.	.	.	.	.	.	.	.	T	.	.	.	.	.	.	.	.	.	.	1		3	1
	311	.	.	.	.	.	.	.	.	.	T	.	.	.	.	.	.	.	.	.	.	4	5	1	5
	307	.	.	.	.	.	.	.	.	.	T	.	.	.	.	.	.	.	.	.	.	2	2	8	11
	313	.	.	.	.	.	.	.	.	.	T	.	.	.	.	.	.	.	.	.	.			1	1
	319	.	.	.	.	.	.	.	.	.	T	.	.	.	.	.	.	.	.	.	.			1	1
1	7	.	.	C	.	T	T	G	.	T	.	T	T	G	T	.	.	A	.	T	.			1	1
2	8	.	.	C	.	T	T	G	.	T	T	T	T	G	T	.	.	A	.	T	.			2	2
4	9	.	.	C	.	T	T	G	.	T	T	T	T	G	T	.	.	A	.	T	.			3	3
5	23	.	.	C	.	T	T	G	.	T	.	T	T	G	T	.	.	A	.	T	.			1	1

*Peptide group #301 is subdivided in 4 parts (A, B, C and D) according to synonymous mutations. **SW = Surface water, DM = Domesticated Mammals, P = Poultry.

**Figure 2:**
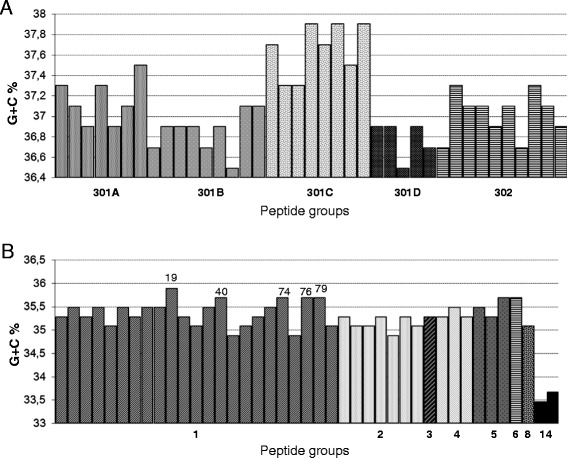
**Percentage of GC contents in nucleotide sequences of** ***gyrA*** **alleles arranged by peptide groups. (A)** *C. coli* **(B)** *C. jejuni*. Numbers of nucleotide alleles are displayed above the bars for values > 35.5% in PG#1.

Figure 2 shows the GC contents of the nucleotide sequences arranged by PGs. Variations in base composition can be observed. A significantly higher GC content (unpaired t-test, p < 0.001) was found in PG #301C from *C. coli* (average = 37.65%, SD = 0.26) compared to the other two groups PG #301B and PG #301D (average = 36.83%, SD = 0.19). By contrast, alleles from the *C. jejuni* species appear more homogeneous in their base contents. The overall average was of 35.33% (SD = 0.25) when excluding PG #14, which displays the lowest level recorded in the *gyrA* sequences (average = 33.57%, SD = 0.14; p < 0.001).

### Distribution of *gyrA* alleles by source

The collection of strains used in this study originated from three sources: surface waters (SW), domestic mammals (DM) and poultry (P). Regarding the *C. jejuni* collection, PG #1 is the largest group, including 23 nucleotide alleles corresponding to more than 50% of the alleles identified for this species (Table 1). However, data could be subdivided in two main sets: (i) the alleles #1, 4, 5 and 7 were commonly identified from the 3 sources (N = 76 for SW, N = 61 for DM and N = 54 for P); (ii) 16 alleles were shared by 105 strains predominantly from environmental source (N = 90 i.e. 43.7% of the SW collection). Within this latest set, the synonymous substitution G408A in nucleotide sequences was never identified from poultry strains. PG #2 is encoded by alleles mainly identified from animal sources represented by 23.3%, 20.2% and 12.6% of the P, DM and SW collections respectively. The PGs #3, 4, 5 and 8 share the synonymous substitution A64G in their nucleotide alleles, significantly associated with poultry source (unpaired t-test, *P* < 0.001). Finally, the only strain harboring an allele specific of the *C. coli* species was isolated from poultry.

The distribution of the *C. coli* strains within PGs previously defined could be summarized as follows: all the strains (N = 77 except one) classified in PG #301B, C and D were isolated from environmental samples (62.2% of the SW collection); poultry strains predominate in PG #302 (N = 84 i.e. 63.1% of the P collection), while all quinolone-sensitive mammal strains were assigned to PG #301A (N = 33 i.e. 71.7% of the DM collection). The seven strains harboring a “*C. jejuni*-like allele” all originate from poultry (Table 2).

### Genotype diversity within the *C. jejuni* collection

**Figure 3:**
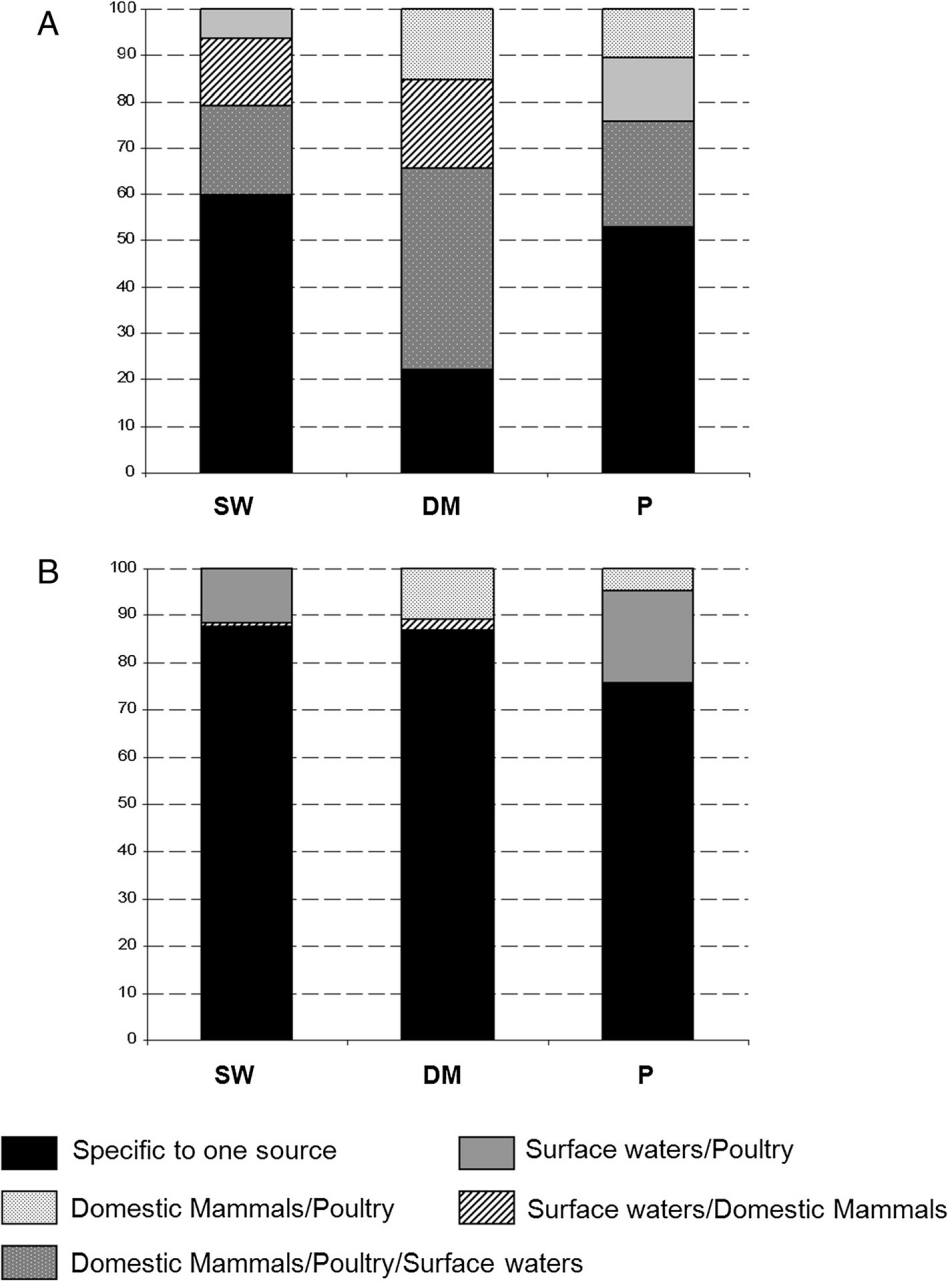
**Distribution of genotypes (ST +** ***gyrA*** **) by source. (A)** *C. jejuni* collection, **(B)** *C. coli* collection. SW = Surface waters, DM = Domesticated Mammals, P = Poultry.

All the strains from this study were further characterized by MLST. For the *C. jejuni* isolates, a total of 170 different STs were identified. Combining MLST with *gyrA* yielded 191 distinct genotypes. The Simpson’s Index of Diversity (SID) was 0.911 (95% confidence intervals (CI) 0.899–0.923) for *gyrA* alleles only, 0.979 (95% CI 0.974–0.984) for MLST only and 0.984 (95% CI 0.979-0.988) for the combination of MLST and *gyrA*. The indexes of association I_A_ calculated for each source using a single representative of each genotype, appeared low and fairly similar, suggesting that each of these populations was highly diverse by recombining to some degree: 0.22 (SW), 0.28 (DM) and 0.19 (P). Population differentiation estimated by the *F*_ST_ values was highest between SW and DM (0.07787, *P* <0.00001), followed by DM and P (0.04074, *P* <0.00001) and lowest for SW and P (0.03476, *P* <0.00001). Nearly half of the strains from the DM set (43.4%), 18.9% of the SW set and 23.2% of the P set had genotypes identified in all three sources (Figure 3A). In the same way, 60.2%, 22.2% and 52.8% of the strains had genotypes specific to SW, DM and P origins, respectively. Finally, 14.6% and 6.3% of the environmental (SW) collection had genotypes common to DM and P sets, respectively. Genotypes not recovered from SW and common to both animal sets represented 15.1% and 10.4% of the DM and P collections, respectively.

### Genotype diversity within the *C. coli* collection

Among the *C. coli* isolates, a total of 146 STs were identified and yielded 194 distinct genotypes when combined with the *gyrA* locus. The SID value for the combined methods was of 0.994 (0.992 – 0.996) versus 0.987 (0.984 – 0.991) for MLST alone or 0.945 (0.936 – 0.953) for the *gyrA* data alone. The I_A_ determined from the SW collection had a value similar to those previously calculated from the *C. jejuni* sets (0.26). In contrast, the I_A_ values from each of the animal population displayed a trend closer to zero indicating a random association between alleles of the 8 loci (i.e. in proximity to linkage equilibrium) by freely recombining (I_A_ for DM = 0.03 and I_A_ for P = 0.05). The population pairwise *F*_STs_ approach generated 3 similar values for each pair combination: SW/DM (0.16295, *P* <0.00001); SW/P (0.16455, *P* <0.00001) and DM/P (0.15848, *P* <0.00001). None of the genotypes was common to all three collections of strains as shown in Figure 3B. However, 87.8%, 87% and 76% of the strains had genotypes specific to SW, DM and P sources, respectively. In the environmental collection, 0.8% and 11.4% of the strains had genotypes common to DM and P sets, respectively. The genotypes recovered only in both animal sources represented 10.9% and 4.5% of the DM and P sets, respectively.

### Quinolone resistant isolates as defined by the C257T mutation

Overall, 43.4% and 17.4% of *C. coli* and *C. jejuni,* respectively, were classified as resistant to quinolones according to the C257T mutation (i.e. the peptide shift Thr86Ile). Quinolone resistance was significantly higher in isolates of poultry origin (*P* < 0.001) for both *C. coli* (67.9%) and *C. jejuni* (38.7%). By comparison, 22.7% and 16.7% of the isolates (including both species) originating from the domestic mammals and surface waters, respectively, were quinolone-resistant.

## Discussion

Sequencing of *gyrA* indicated that this locus was informative in several different ways for characterizing *Campylobacter* isolates. First, the alleles of the 496 nucleotide fragments were suitably different in sequence identity between *C. jejuni* and C. *coli* to be assigned to one or the other of these species. The distribution of these alleles confirmed that recombination events between species occur rather infrequently and in an asymmetric gene flow [33]: one *C. jejuni* had a typical *C. coli* allele whereas 4 *C. coli* had a typical *C. jejuni* allele. Two other studies using PCR and sequencing data targeting *gyrA* also identified a *C. jejuni* segment within a *C. coli* isolate [34,35], supporting previous findings that gene flow is rather unidirectional from *C. jejuni* to *C. coli* [33,36].

Sequencing of *gyrA* revealed a similar population structure as that obtained by MLST or rMLST (Ribosomal Multilocus Sequence Typing, [37]). In particular, the phylogenetic analysis clearly organized *C. coli* into 3 distinct clades as previously described by Sheppard *et al*. [33,36] (Figure 1). Furthermore, peptide groups 301A and 302 in our study (Table 2) contain alleles commonly found in domestic animals, and they correspond to the agricultural *C. coli* lineage of the evolutionary scenario proposed by Sheppard *et al*. [38]. In addition, peptide groups 301B and 301C (Table 2) match with the clades 2 and 3 observed by Sheppard *et al.* [38] including only alleles recovered from environmental isolates, i.e. from surface waters in our study. In contrast to *C. jejuni*, the *C. coli* assigned alleles are predominated by synonymous mutations. As a result, the peptide group 301C is characterized by alleles with a higher GC content (Figure 2A) generated by nucleotide changes only located in the third positions of codons. This trend was also reflected in genotypes linked to this peptide group 301C i.e. by compiling GC content from the internal fragments of the 7 MLST housekeeping genes with the *gyrA* alleles (a total of 3,805 bp in length, see Additional file 1). This kind of GC rich version of genes, independent of adaptive codon usage was significantly associated with effects on bacterial fitness, which could be explained by higher stability of mRNAs [39]. The study of Foerstner *et al.* [40] linked the genomic GC pattern of bacterial populations to environmental factors like ultraviolet irradiation as an example. Thus, the difference in synonymous GC contents found in the *gyrA* alleles from the peptide groups 301B and 301C, suggests that these lineages originated from two distinct but not yet identified ecological niches. By using concatenated nucleotide sequences from MLST data, isolates from our *gyrA* peptide group 301B would be classified in the clade 2 from the study of Colles *et al.* [41] (see Additional file 2) including the majority of the STs identified from wild Mallard ducks. Among our collection of surface water isolates, we similarly observed three clades: one associated with domestic animals and the other two of wildlife origin, one of which potentially linked to waterfowl. Nevertheless, with a more discriminative approach based on genotypes defined by combining the 7 housekeeping genes from MLST with the *gyrA*, the populations of *C. coli* displayed a high specificity in their distribution by sources (Figure 3). None of the 194 genotypes identified was found in all three collections (SW, DM and P) and *F*_STs_ values calculated by pair comparisons were about 4 times higher than those computed from *C. jejuni* pairs. The fact that domesticated mammal isolates were poorly represented in our environmental samples could have resulted from a temporal and geographic sampling bias. Half of the collection was mainly isolated in 2006 [3] and the other half was collected from distant geographic locations. As to the isolates originating from poultry, it must be emphasized here that domestic production of broilers is negligible and there is no poultry hatchery in the country. Thus, direct contamination of environmental waters by local poultry farms is largely restricted.

Regarding the *C. jejuni gyrA* sequences, two lineages were clearly distinguished (Figure 1). One branch is represented by the peptide group #14, encoded by the alleles #54 and #55 recovered from surface waters isolates only. These nucleotide sequences are again mainly differentiated by their GC content, but this time, below the mean of each of the other groups (Figure 2). The two STs associated with these strains are newly described (ST 5841 and ST 6171) and correspond to variants of *a C. jejuni* clone associated with bank voles [42]. Interestingly, these strains also displayed atypical profiles with the duplex-real time PCR implemented in this study for identifying isolates at the species level. An extra PCR was needed to confirm the presence of the *hipO* gene (see the Methods section). In summary, this phylogenetic lineage originated from a wildlife source, whereas the other one is composed of *gyrA* alleles mainly shared by domesticated mammals. However, the peptide group #1 from the main branch which is encoded by the largest number of alleles (N = 23), could be subdivided into two sets of sub-clusters: one set harboring strains isolated from domestic mammals (N = 9) and the other set being highly specific to environmental samples (N = 14). From this last set, five sequences (#19, 40, 74, 76 and 79) display a slightly higher GC content (Figure 2B) as a potential “trace signature” of different ecological niches. In addition, within this same peptide group #1, the nucleotide alleles with the synonymous substitution G408A (#11, 39, 40, 41, 56, 66 and 79) were never recovered from poultry strains. This change is also present in alleles from peptide group #14 previously discussed and linked to small mammals [42]. The most obvious host signature established in our study is the non-synonymous substitution A64G corresponding to the change Ser22Gly in the amino acid sequence. This point mutation was previously observed by Ge *et al.* [43] in a study on antimicrobial resistance of strains isolated from poultry meat in which 76.2% ciprofloxacin-resistant *C. jejuni* harbored this particular substitution in their *gyrA* sequence (N = 42). Jesse *et al.* [44] also noticed this mutation in isolates from chicken and turkeys and suggested that it does not contribute to quinolone resistance but may be indicative of *gyrA* alleles predominantly found in poultry. Our results confirm this finding: 11 isolates with the Ser22Gly but without the Thr86Ile substitution were classified as susceptible to quinolones according to the cut off values recommended by the European commission [45] (see Additional file 3). Also, peptide groups #3, 4, 5 and 8 with this particular change on codon 22, are significantly associated with poultry source (*P* = 0.001). This host signature could be used as a specific molecular marker of domestic birds.

Our study also found that quinolone resistance was higher in isolates originating from poultry than from other sources. Recently, Han *et al.* [46] demonstrated that this particular mutation generates a fitness advantage for *Campylobacter* in chicken through a reduced supercoiling activity of the GyrA enzyme. As DNA supercoiling is directly involved in gene expression, their findings suggested that the altered function of the enzyme modulates the fitness of resistant strains whose prevalence persists in poultry production even in the absence of fluoroquinolone use. The European report on antimicrobial resistance in zoonotic bacteria [12] reported very high fluoroquinolone resistance levels in *Campylobacter* isolated from broilers (76%) and broiler meat (58%). Our results concur with the report in that resistance levels vary substantially in different hosts.

## Conclusion

The interest of the sequence-based method described herein targeting the gyrase subunit A lies not only in providing information on quinolone resistance but also on strain origin. As the gyrase is an essential gene for bacterial viability and also plays a role in gene expression, some patterns in sequences appear to be informative as potential host signature, predictive of particular ecological niches. All the sequences of alleles defined here are freely accessible on the website of the *Campylobacter* MLST website (http://pubmlst.org/campylobacter/) developed by Keith Jolley and sited at the University of Oxford [47]. We believe that this tool could be useful for basic surveillance of campylobacteriosis in two ways. For long-term surveillance, it could be combined with MLST data for increased discrimination power, and would help in identifying source attribution of ST complexes shared by more than one sample population: ST21, ST45 and ST48 complexes for example [48]. For short term surveillance *i.e.* detection of temporal clusters of human cases, it could provide some indication on the potential infection source involved when combined with *porA* or *flaA* typing [8].

## Additional files

Additional file 1:
**GC contents using concatenated nucleotide sequence: 7 housekeeping genes from MLST with** ***gyrA*** **alleles (3805 bp).** Results from the 187 genotypes are classified according *gyrA* peptide groups. Average in GC% for each group are shown. Additional file 2:
**Neighbour-joining radial distance phylogenetic tree constructed with concatenated nucleotide sequences from STs identified from this study and from Colles** ***et al.*** [41] **on wild and domesticated ducks.**
Additional file 3:
**MICs recorded for** ***C. jejuni*** **isolates with Ser22Gly but without the Thr86Ile substitution.** Interpretative thresholds for resistance (R): CIP_R >0.5 and NAL_R > 16.
