# Supplementary material for: Investigating the host specificity of Campylobacter jejuni and Campylobacter coli by sequencing gyrase subunit A
Source: BMC Microbiol. 2014 Aug 28;14:205. doi: 10.1186/s12866-014-0205-7 (PMC4156964; doi:10.1186/s12866-014-0205-7)
Supplement: Additional file 1: — GC contents using concatenated nucleotide sequence: 7 housekeeping genes from MLST with gyrA alleles (3805 bp). Results from the 187 genotypes are classified according gyrA peptide groups. Average in GC% for each group are shown. [file 12866_2014_205_MOESM1_ESM.pdf]

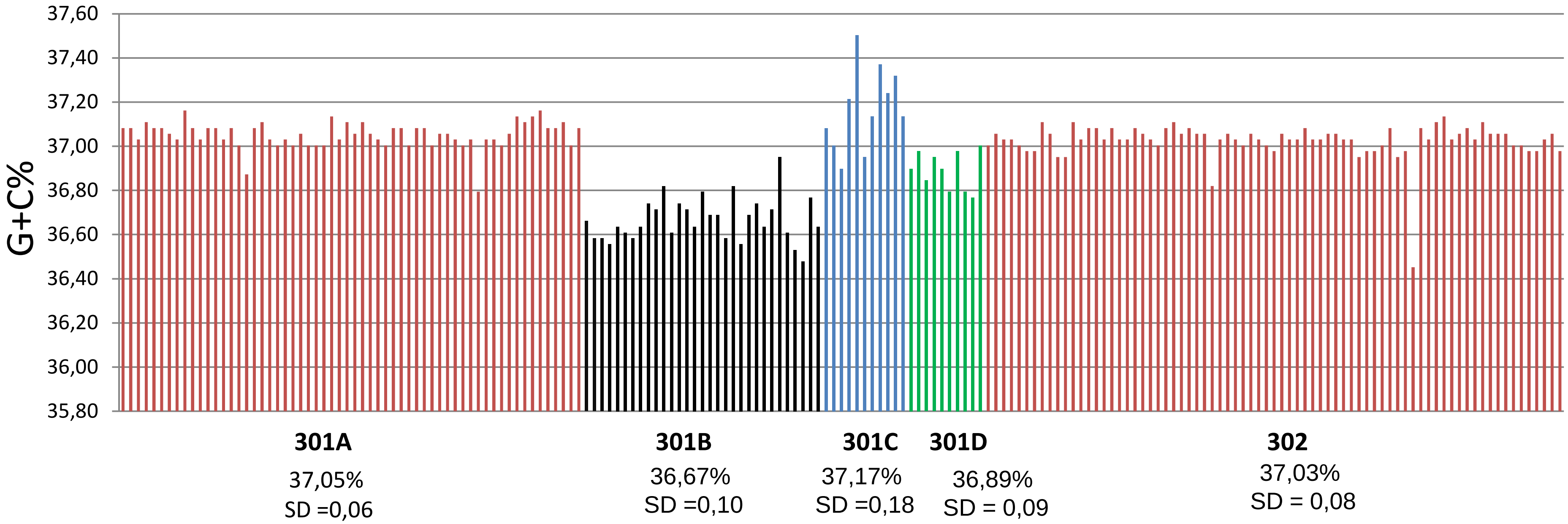

Additional file 1

GC contents using concatenated nucleotide sequence: 7 housekeeping genes from MLST with *gyrA* alleles (3805 bp). Results from the 187 genotypes are classified according *gyrA* peptide groups. Average in GC% for each group are shown.
