# Supplementary material for: Investigating the host specificity of Campylobacter jejuni and Campylobacter coli by sequencing gyrase subunit A
Source: BMC Microbiol. 2014 Aug 28;14:205. doi: 10.1186/s12866-014-0205-7 (PMC4156964; doi:10.1186/s12866-014-0205-7)
Supplement: Additional file 2: — Neighbour-joining radial distance phylogenetic tree constructed with concatenated nucleotide sequences from STs identified from this study and from Colles et al. [41] on wild and domesticated ducks. [file 12866_2014_205_MOESM2_ESM.pdf]

Additional file 2
Neighbour-joining radial distance phylogenetic tree constructed with concatenated nucleotide sequences from STs identified from this study and from Colles *et al.* (2011) on wild and domesticated ducks.

Wild ducks (Colles *et al.* 2011)
Surface waters (this study)
Domesticated animals and surface waters (this study)

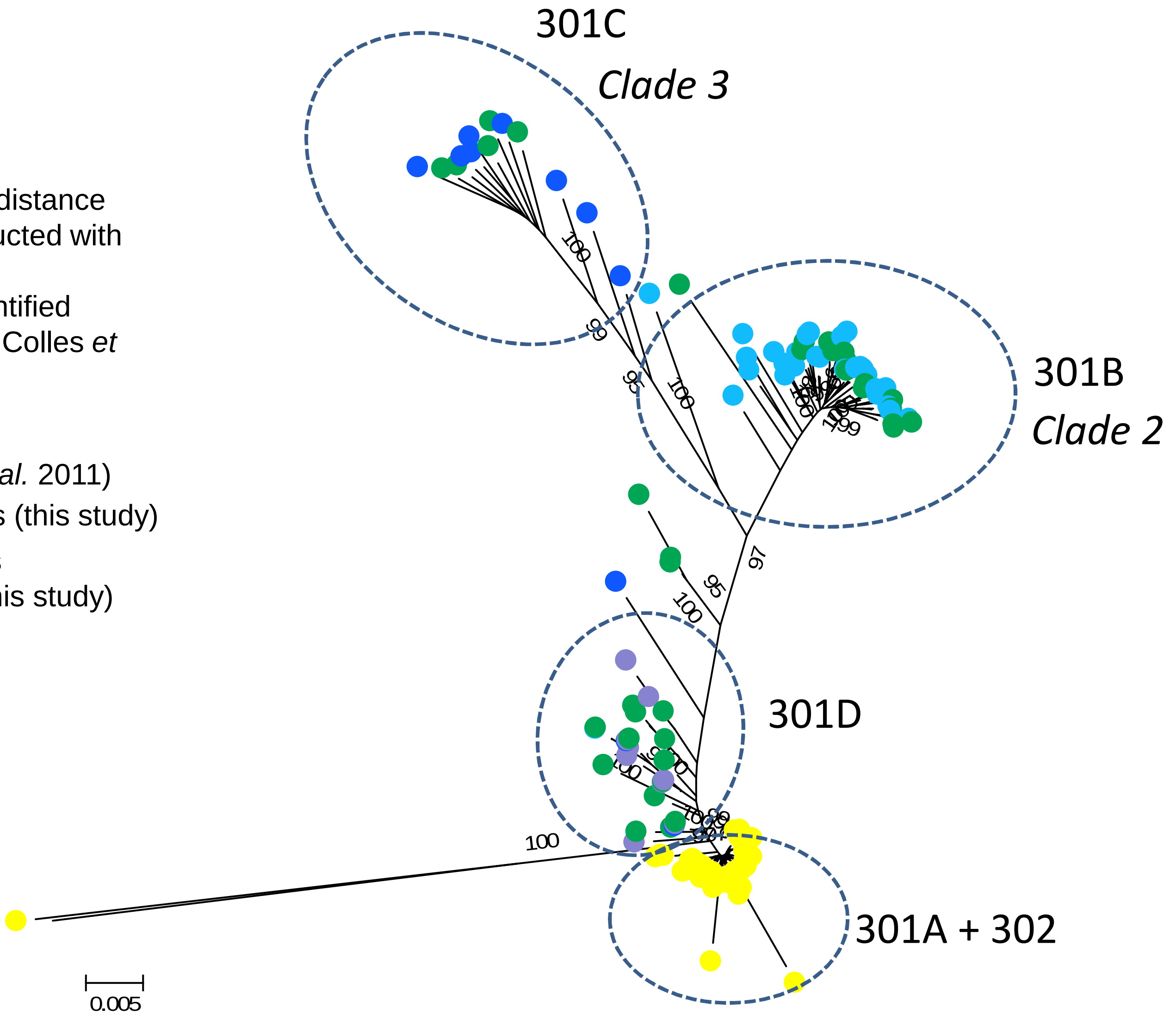

Bootstrap support values (%) for each of the nodes leading to the *gyrA* sequence clusters are indicated.
