# Supplementary material for: Investigating the host specificity of Campylobacter jejuni and Campylobacter coli by sequencing gyrase subunit A
Source: BMC Microbiol. 2014 Aug 28;14:205. doi: 10.1186/s12866-014-0205-7 (PMC4156964; doi:10.1186/s12866-014-0205-7)
Supplement: Additional file 3: — MICs recorded for C. jejuni isolates with Ser22Gly but without the Thr86Ile substitution. Interpretative thresholds for resistance (R): CIP_R >0.5 and NAL_R > 16. [file 12866_2014_205_MOESM3_ESM.pdf]

Additional file 3: MICs recorded for *C. jejuni* isolates with Ser22Gly but without the Thr86Ile substitution. Interpretative thresholds for resistance (R): CIP_R >0.5 and NAL_R > 16

Isolate	Source	*gyrA* allele no	MIC mg l^{-1}	
			CIP	NAL
C111066	chicken	6	0.064	<0.5*
C100198	chicken	10	0.064	2
C100199	chicken	10	0.064	1
C110443	chicken	10	0.032	<0.5*
C120384	cattle	10	0.032	<0.0625*
C121822	cattle	10	0.064	1
E60476P	cattle	10	0.047	3
E60735P	cattle	10	0.064	3
E70030	chicken	10	0.064	2
E70167	chicken	10	0.032	8
C120911	chicken	23	0.125	<4*

CIP, ciprofloxacin; NAL, nalidixic acid

* MICs values deduced from disk diffusion assay – all other values were obtained by E.test method.
